# Knee Osteoarthritis—How Close Are We to Disease-Modifying Treatment: Emphasis on Metabolic Type Knee Osteoarthritis

**DOI:** 10.3390/life13010140

**Published:** 2023-01-04

**Authors:** Sevdalina Nikolova Lambova

**Affiliations:** 1 Department of Propaedeutics of Internal Diseases, Faculty of Medicine, Medical University of Plovdiv, 4002 Plovdiv, Bulgaria; sevdalina_n@abv.bg; 2 Department in Rheumatology, MHAT “Sveti Mina”, 4002 Plovdiv, Bulgaria

Osteoarthritis (OA) is a whole-joint disease that affects cartilage, bone, and synovium as well as ligaments, menisci, and muscles. Although previously considered to be a non-inflammatory disease, it is now widely established that low-grade inflammation plays a pivotal role in the disease pathogenesis. Although the low-grade inflammation in OA may be not the primary disease trigger, it might be involved in disease progression [1]. The heterogeneous nature of OA regarding localization and its dominant pathogenic mechanism are the major causes for unsatisfactory therapeutic results in relation to slowing of structural progression. The standard pharmacological treatments used in OA are non-steroidal anti-inflammatory drugs, analgesics, and symptomatic, slow-acting agents with chondroprotective properties (e.g., glucosamine, chondroitin, soy and avocado, and intra-articular hyaluronic acid) [2,3].

The knee is the most commonly affected joint in OA. The existence of different phenotypes of knee OA has been suggested; however, the precise criteria for their classification are not well-defined. While clinical phenotypes are characterized by common risk factors and can be used to determine progression and predict therapeutic response, the endotypes are disease subtypes characterized by well-defined molecular mechanisms, i.e., cellular and biochemical signaling pathways [4].

Based on a systematic literature review, Dell’Isola et al. (2016) have proposed the existence of six phenotypes of knee OA related to predominant pathogenic mechanisms, i.e., a chronic pain phenotype, an inflammatory phenotype, phenotypes associated with alterations in bone and cartilage metabolism, with metabolic syndrome, a mechanical phenotype, and minimal joint disease. The chronic pain phenotype is thought to be related to central sensitization and alterations in pain neurophysiology and the psychological profile. Regarding the inflammatory type of knee OA, gene overexpression of inflammatory cytokines was detected, e.g., interleukin (IL)-1β, cyclooxygenase 2, and macrophage-inflammatory proteins. Higher level of pain and faster radiographic progression were observed in these cases compared to those with low cytokine expression. In the metabolic type of knee OA, it has been suggested that metabolic syndrome contributes to the development of knee OA, and this phenotype has been associated with higher levels of leptin and high-sensitivity CRP (hsCRP). Mechanical overload and previous injuries lead to development of the mechanical type of knee OA. Minimal joint disease is characterized by mild degeneration, mild clinical symptoms, and a slow progression rate. Considering the variables used to identify different phenotypes, the authors have concluded that they probably do not exist as distinct clinical entities and that an overlap between different subtypes is possible. Thus, the determination of the presence of such an overlap between different phenotypes may provide clues for the selection of appropriate combined therapeutic strategies [5]. In a systematic review, Deveza et al. (2017) have aimed to identify characteristics of knee OA that may be used to classify the diseases of mutually exclusive phenotypes. Accordingly, potential definitions of clinical, laboratory, and imaging phenotypes were summarized. Psychological factors including depression, a patient’s pain sensitivity profile, catastrophizing and coping behaviors were most frequently used in the analyzed studies to define the clinical phenotypes. Presence of metabolic syndrome and obesity was also considered in some sources. In a part of the studies laboratory phenotypes have been defined based on biochemical markers [6]. The presence of inflammation in knee OA evaluated via hsCRP measurement was associated with higher values of the Western Ontario and McMaster Universities (WOMAC) scores and body mass index (BMI) [7]. In 111 patients with symptomatic knee OA, plasma levels of IL-1 receptor antagonist (IL1Ra) were modestly associated with disease severity and progression [8]. In 117 patients with knee OA, Berry PA et al. (2010) have assessed the levels of the biomarkers of cartilage metabolism (cartilage oligomeric matrix protein, N-propeptide of type IIA procollagen, and type II collagen breakdown product/collagen type-II cleavage) as predictors of cartilage volume loss measured via magnetic resonance imaging (MRI) over 2 years. In the subgroup of patients with low levels of cartilage oligomeric matrix protein and N-propeptide of type IIA procollagen, a significantly lower rate of medial cartilage volume loss was observed. The level of collagen type-II cleavage was not significantly associated with the rate of cartilage volume loss [9]. Berry PA et al. (2010) also evaluated the association between markers for bone resorption (N-telopeptide of type I collagen and C-telopeptide of type I collagen) and bone formation (intact N-terminal propeptide of type I procollagen and osteocalcin) and cartilage volume loss using MRI (117 patients with knee OA; 2 year-follow-up). Significant associations between the ratios of the markers of bone formation to resorption and cartilage loss were not present at baseline. A further analysis of the patients with high levels of N-terminal propeptide of type I procollagen and osteocalcin showed that the increasing levels of bone resorption markers (N-telopeptide of type I collagen and C-telopeptide of type I collagen) were significantly correlated with reduced cartilage loss. However, in the subgroups with low levels of bone formation markers, significant associations between the markers of bone resorption and cartilage loss were not detected. Thus, it was concluded that a higher level of bone remodeling reflected by a higher serum level of the markers of bone formation and resorption may indicate better prognosis in knee OA patients [10]. Adverse structural outcomes with a higher joint-space-narrowing rate over 2 years was observed in cases of cytokine overexpression along with worse radiographic severity in the phenotype with higher levels of hsCRP. The criteria for different imaging phenotypes have been investigated with respect to the predominant knee compartment affected and the cartilage damage profile (assessed as denuded bone areas on MRI). Knee pain was observed more frequently in patients with centrally located denuded bone areas [6].

## Metabolic Type Knee Osteoarthritis

The association between the presence of metabolic syndrome and OA is observed not only for the knee joint, where mechanical overload plays a role, but also for hand OA, which suggests the involvement of systemic factors. Both metabolic syndrome and OA are associated with low-grade inflammation and there are data demonstrating that metabolic syndrome may contribute to the development of OA. In obesity-associated knee OA, systemic factors are thought to play a significant role alongside the higher biomechanical load. The systemic effects of obesity on the development of OA are likely mediated by the action of adipokines, insulin resistance, abnormal levels of microRNAs in the context of low-grade systemic inflammation. Regarding the accompanying pathological conditions in the scope of the metabolic syndrome, there are hypotheses concerning the presence of an association between dyslipidemia and OA. In addition, arterial hypertension might lead to vascular alterations in the synovium and subchondral bone that may contribute to changes in synovial fluid, cartilage metabolism, pathological remodeling of the subchondral bone, and the initiation of OA [11]. In a meta-analysis conducted by Wang et al. (2016), which included 3202 cases with knee OA and 20968 controls, it was concluded that metabolic syndrome increases the risk of developing knee OA after adjustment for many risk factors, such as age, sex, BMI, physical activity, etc. [12]. In an own study, among 73 patients with symptomatic primary knee OA (43 with accompanying obesity and BMI ≥ 30 kg/m^2^ and 30 patients with BMI < 30 kg/m^2^, from 2nd to 4th radiological stage according to Kellgren–Lawrence scale, and aged between 35 and 87 years (mean age 66 years), significantly higher leptin and resistin levels were found in cases with OA and obesity compared to healthy subjects. Interestingly, serum leptin levels were correlated with the radiological stage of the disease, i.e., higher levels were present in cases with more advanced structural changes (radiological stages of III and IV). Novel clinical correlations were observed in patients with isolated knee OA who were significantly younger and had higher BMI compared to the patients who had also OA with other localization (spondylarthritis, hip OA, and generalized OA) [13]. Although the age above 50 years is an established classification criterion for knee OA, recent studies report an increase in the incidence of the disease in patients below 40 years [14]. In patients with isolated knee OA, significantly higher leptin and resistin levels were found in comparison with cases with OA combined with involvement of other joints. These data support the hypothesis that the presence of obesity leads to earlier development of knee OA that precedes the appearance of osteoarthritic processes with other localization, as well as the notion that metabolic knee OA represents a specific phenotype of the disease whose classification criteria are to be precisely defined. Consequently, metabolic knee OA is emerging as a distinct clinical phenotype that may be considered as an additional musculoskeletal component of metabolic syndrome. It is thought to occur earlier in younger patients with obesity and is associated with increased adipokine levels [13].

Interestingly, a comparison of the MRI features regarding metabolic knee OA and knee OA related to physical activity in lean patients (two groups of fifty subjects) has shown significant differences concerning the scores for cartilage damage, which were higher in the patella, trochlea, and lateral femur in patients with metabolic knee OA. Osteophyte scores were also higher for all compartments in metabolic knee OA, with statistically significant differences for the patella, trochlea, and medial tibia [15].

The research on the precise definition of different phenotypes of knee OA is ongoing. Clinical, laboratory, and imaging parameters may be used in this regard. The combined assessment of adipokine levels, markers of cartilage and bone metabolism, and highly sensitive markers of inflammation, together with clinical and imaging findings, would facilitate the better classification of the metabolic type of knee OA.

## Future Directions Regarding Therapeutic Strategies for Osteoarthritis

Currently, there is no approved disease-modifying drug for OA. This is related to the heterogeneous nature of the disease with respect to its pathogenesis and localization [16]. In addition, cartilage is difficult to be repaired being an avascular structure. Based on the hypothesized existence of distinct subtypes of knee OA, Oo et al. suggested the benefit of an endotype-oriented summary of the current research in the field of future therapies according to the primary target of the interventions in three major subtypes of the disease i.e., cartilage-, bone-, and synovium-driven endotype [4]. Future precise definitions of different endotypes of OA with different localizations would facilitate the selection of appropriate therapeutic strategies, which may also include drug combinations, considering the complex pathogenesis of the disease, and potentially lead to the determination of successful disease-modifying interventions in the different disease subtypes.

The topics of current interest regarding the *cartilage-driven endotype* of OA include proteinases inhibitors, recombinant fibroblast growth factor, Wnt-signaling inhibitors, gene-therapy that promotes transforming growth factor-β transcription, and senolytic therapies that attempt to influence senescence, i.e., altered age-related response to stress and the arrest of cell proliferation. The evaluation of drugs that influence bone remodeling in the *bone-driven endotype* is ongoing. The most abundant data are available for bisphosphonates. Diacerein, TNF-α and IL-6 inhibitors, the anti-inflammatory cytokine IL-10 are subjects of research for the *synovitis-driven endotype* [4].

A significant difference in the gene expression patterns between osteophytic and articular chondrocytes has been detected in in vitro studies of knee joint cartilage with pronounced expression of antagonists inhibiting bone morphogenetic protein and the Wnt-pathway in articular cartilage that might stabilize the chondrocyte phenotype and prevent their terminal differentiation and endochondral bone formation [17]. The *Wnt signaling pathway* is involved in the regulation of progenitor cell differentiation, cartilage and bone metabolism, and inflammatory responses in the knee joint. Lorecivivint leads to the inhibition of the Wnt pathway and inflammation, and its intraarticular administration (optimal dose 0.07 mg) led to clinically significant improvement in a 24-week study, but without providing a difference in terms of the mean joint space width. An evaluation of the structure-modifying properties of lorecivivint should be performed in clinical trials with a longer duration [16].

*Recombinant Fibroblast Growth Factor 18 (Sprifermin)* stimulates chondrocyte proliferation and extracellular matrix synthesis. In a 5-year, placebo-controlled, clinical trial that included 549 patients with symptomatic knee OA (radiographic stage 2nd and 3rd according to the Kellgren–Lawrence scale), an intra-articular administration of 100 μg of sprifermin every 6 or 12 months led to significant increases in total femorotibial cartilage thickness vs placebo after 2 years, while there was no significant difference for the dose of 30 μg every 6 or 12 months [18].

Interestingly, in an animal model of OA, bone loss and decreased bone density has been found in the early stages of the disease, which includes both thinning of the subchondral bone plate and the increased porosity of the subchondral trabecular bone. In the next stages, during which there are radiological signs of subchondral sclerosis, increased collagen synthesis with poor mineralization and bone cysts could be found in the subchondral bone [19,20]. Thus, it has been hypothesized that the targeting of bone could be an effective treatment for symptomatic relief and slowing structural progression in knee OA [20]. The observations concerning the role of bisphosphonates in knee OA are inconclusive. In a meta-analysis by Vaysbrot et al. (2018) that included 3013 patients (2767 of whom were on oral risedronate), it was found that bisphosphonates do not provide symptomatic relief and do not slow radiographic progression in knee OA. However, it has been suggested that biphosphonates may be beneficial in knee OA patients who exhibit a high subchondral bone turnover rate [21]. In a small, placebo-controlled trial including 59 patients with knee OA and bone marrow lesions detected via MRI that received either 5 mg of zolendronic acid or placebo, a significant reduction in the VAS pain score was registered after 6 months, and the difference was no longer significant after 12 months. A significant reduction in bone marrow lesions was also documented after 6 months after adjusting for age, sex, pain score, and drug use. After a 12-month follow-up, the tendency for a reduction in bone marrow lesions persisted but with borderline significance [22]. In a recent systematic review that included both preclinical and clinical studies, it was concluded that bisphosphonates exhibit chondroprotective effects in animal models of OA, wherein zolendronic acid produced the greatest effect. The analysis of the data from the clinical trials showed that biphosphonates reduce osteoarthritic changes in a dose-dependent manner, providing superior chondroprotective properties at higher doses. Their efficacy also likely depends on the time of their administration during the disease course. Bisphosphonates have demonstrated a chondroprotective effect that is highly variable in different studies, as well as an anti-inflammatory effect in the synovial membrane without the suppression of osteophyte formation. Evidence regarding the duration of the treatment is lacking [23]. Current data concerning the efficacy of anti-osteoporotic drugs towards OA are inconsistent and require further research, especially with respect to patients with OA and high bone turnover [4] or as a component of a combined therapeutic strategy. In this regard, reliable biomarkers should be determined to select the patients appropriate for this treatment approach.

Synovitis is a common feature of OA and is characterized by synovial hyperplasia, fibrosis, vascularization, and effusion. Ultrasound and MRI are used to confirm the presence of synovitis, but it remains unclear whether its presence indicates disease activity or a distinct subtype of OA. Macrophages and T-lymphocytes are the predominant immune cells in OA synovitis and several proinflammatory cytokines are involved in disease pathogenesis with leading roles of IL-1β and TNF-α. In addition, it has been suggested that synovitis may indicate disease progression [24]. Persson et al. (2018) evaluated the efficacy of conventional and biological disease-modifying, anti-rheumatic drugs (DMARDs) in OA in a meta-analysis that included 11 randomized clinical trials with 1205 participants (757 patients on conventional DMARDs and 448 cases on biologics). Five trials included patients with knee OA and one clinical study included patients with clinical signs of synovitis. The overall results showed that DMARDs were statistically superior to placebo with respect to pain relief. However, the effect size was 0.18, which is not clinically significant (the threshold for effect size is 0.5). A separate assessment of conventional and biologic DMARDs found that both groups were not superior to placebo in terms of pain relief. No difference was detected between hand and knee OA. The poor efficacy of conventional and biologic DMARDs is explained by the fact that inflammation might not be the primary trigger for OA pain [25] or by the improper selection of appropriate candidates for such a treatment due to the absence of established criteria from imaging and laboratory biomarkers [24]. In a recent meta-analysis by Li et al. (2022), the efficacy of different biologics (TNF-α blockers as well as IL-1, IL-6, and IL-17 inhibitors) was assessed. The authors analyzed 15 studies that included 1566 patients with knee and hand OA. The routes of drug administration were variable, including intra-articular. The authors concluded that infliximab may relieve pain to a greater extent than other biologics, while the other assessed biological agents did not lead to improvement of physical function and stiffness compared to placebo. However, the data should be confirmed in future studies as the conclusion regarding infliximab was drawn based on the results of two studies that incorporated 26 patients and combined data with different doses, routes of administration, and localizations of OA (hand and knee OA). The authors hypothesized that females and inflammatory phenotypes of OA with the presence of synovitis and effusion could be potential candidates for treatment with infliximab [26].

Mesenchymal stem cells have been extensively studied as a therapeutic option in OA due to their ability to differentiate into chondrocytes, which may facilitate cartilage regeneration, as well as for their immunomodulatory properties [27]. Centeno et al. (2010) have reported >50% reduction in symptoms in 135 patients with knee OA (a therapeutic response was observed in two thirds of patients) after being treated with mesenchymal bone marrow stem cells obtained from the iliac bone at an average of just over 11 months post-procedure. Approximately two thirds of the examined patients were candidates for a knee arthroplasty and only 6.9% of the treated knee patients reported that they planned to undergo knee replacement surgery despite the mesenchymal stem cell therapy. Neoplastic or ectopic tissue complications were not documented [28]. Adult human bone marrow-derived, cultured, mesenchymal stromal cells (Stempeucel^®^) showed a significant downregulation of Sox9 in vitro. Sox9 is an early inducer of chondrogenesis and the upregulation of Col2A, the gene that encodes type 2 collagen, which is a major cartilage matrix protein. In an animal model of OA, Stempeucel^®^ induced cartilage repair. In a randomized, double-blind, placebo-controlled, phase II study, the intraarticular administration of Stempeucel^®^ in patients with knee OA led to decreases in pain (VAS and WOMAC) and improved physical activity (WOMAC) [29]. Other sources of stem cells include adipose tissue from different sites, e.g., the buttocks, abdomen, and infrapatellar fat pads. The results regarding the treatment of OA patients with adipose-derived mesenchymal stem cells were summarized in a systematic review by Hurley E. et al. (2018) that included 16 studies. All of the studies prepared adipose-derived stem cells in the form of the stromal vascular fraction, which is a component of lipoaspirate obtained by liposuction and contains, together with stem cells, various other cell types, including pericytes, fibroblasts, preadipocytes, monocytes, macrophages, and red blood cells. Thus, it has been concluded that the reported results do not evaluate the response only to the stem cell fraction. This systematic review showed that the stromal vascular fraction obtained from adipose tissue may produce favorable clinical outcomes that pose a minimal risk of side effects to OA patients. In addition, eight of the sixteen studies also included the radiological outcomes using MRI or X-ray in the reported analyses. Notably, improved cartilage thickness was reported in the majority of the studies using MRI assessment [30].

A novel study conducted by Timmons R. et al. (2022) reported beneficial results in 30 knee OA patients (at the age of 63 ± 10.9 years) after injection with 1 mL of thawed, cryopreserved umbilical cord tissue donated by healthy volunteers with 4 mL saline. Umbilical cord tissue from healthy live births is suggested to possess regenerative, anti-inflammatory, and immunomodulatory properties due to its high concentrations of growth factors, cytokines, and mediators including IL-10, vascular endothelial growth factor, and tissue inhibitors of matrix metalloproteinases. Improved pain and physical function as well as reduced use of opiates and nonsteroidal anti-inflammatory drugs were registered soon after the treatment and continued, in most of the cases, for 24 weeks. For many patients, the improvement continued up to 24 months after a single injection. These observations require confirmation in future double-blinded, randomized studies [31].

## Therapeutic Considerations in Metabolic Knee Osteoarthritis

Considering the lack of disease-modifying treatments for OA, the effect of the drugs used for treating metabolic syndrome on joint homeostasis constitutes an intriguing question. Thus, the choice of combined therapeutic strategies may influence both components of metabolic syndrome and the metabolic type of knee OA. Interestingly, treatment with fenofibrate in in vitro experiments led to reduced proteoglycan loss and protected against cartilage degeneration induced by the administration of IL-1β. In our own study, a histological examination incorporating a mouse model of OA showed decreased cartilage degeneration after treatment with metformin, and the effect was more pronounced in the group treated with a combination of metformin and alendronate [32]. In patients with knee OA and accompanying obesity (BMI ≥ 30 kg/m^2^; ≥2nd radiological grade according to Kellgren–Lawrence scale), metformin administration has been associated with slower cartilage volume loss in the medial compartment of the joint assessed via MRI after 4 years. These results suggest possible long-term beneficial effects and disease-modifying potential of metformin in patients with knee OA and obesity that require confirmation in future clinical trials [33].

By analyzing the effects of different classes of antihypertensive drugs on pain and joint space width among 1945 patients who used antihypertensive drugs from the Osteoarthritis Initiative project, it has been shown that, among females, the users of *calcium channel blockers* exhibited significantly higher pain scores than the groups using beta-blockers and angiotensin receptor blockers. Compared to the common antihypertensive drugs (beta-blockers, ACE inhibitors, angiotensin receptor blockers, and thiazide diuretics), calcium channel blockers were associated with reduced joint space width, higher replacement rates, and higher pain scores [34]. An antinociceptive effect has been demonstrated for the beta blocker bupranolol in a mouse model of chronic pain [35]. Among 873 patients with hip and/or knee OA and hypertension, beta blocker users had a lower prevalence of pain and lower pain scores [36]. Interestingly, in a rat model of OA, captopril demonstrated a chondroprotective effect and local suppression of the renin–angiotensin system [37]. However, upon analysis of the data of the Osteoarthritis Initiative, which included 1796 participants with knee OA who had used ACE-inhibitors and angiotensin receptor blockers, no association was found between the blockade of the renin–angiotensin–aldosterone system and knee pain, function, and OA radiographic progression [38].

Emerging data regarding the existence of metabolic knee OA and the effects of the drugs used for treating different components of metabolic syndrome on joint homeostasis may facilitate the selection of personalized drug combinations in the presence of clinical indications, together with chondroprotectors and local treatment, which, in turn, may lead to the determining of successful disease-modifying therapeutic strategies in OA.
